# Health Professionals’ Perspectives of the Role of Palliative Care During COVID-19: Content Analysis of Articles and Blogs Posted on Twitter

**DOI:** 10.1177/10499091211024202

**Published:** 2021-06-30

**Authors:** Gursharan K. Singh, Joanna Rego, Shirley Chambers, Jennifer Fox

**Affiliations:** 1 Faculty of Health, Centre for Healthcare Transformation, Queensland University of Technology (QUT), Brisbane, Queensland, Australia; 2 Cancer and Palliative Care Outcomes Centre, School of Nursing, Queensland University of Technology (QUT), Brisbane, Queensland, Australia

**Keywords:** qualitative research, palliative care, COVID-19, medical staff, naturalistic data, knowledge and skills

## Abstract

**Objective:**

The COVID-19 pandemic has resulted in health professionals facing unprecedented challenges and changes in practice. Responding to humanitarian crises involves saving lives and reducing suffering and as such, palliative care is an essential component of the response to COVID-19. This study aimed to explore health professionals’ perspectives of the role of palliative care during the COVID-19 pandemic by analyzing their views and experiences expressed in articles and blogs located via Twitter.

**Method:**

Qualitative content analysis of 33 online articles and blogs written by health professionals from 1 February 2020 to 30 June 2020. Online articles and blogs were included if the URL was posted on Twitter. The consolidated criteria for reporting qualitative research (COREQ) guided data collection and reporting.

**Results:**

The health professionals whose viewpoints were analyzed were from Europe, North America, Oceania and Asia. The themes derived from content analysis were (a) the recognized value of palliative care during the pandemic, (b) positioning palliative care as every health professional’s responsibility and (c) specialist palliative care supporting and guiding health professionals

**Conclusions:**

This study utilizes a unique approach to exploring the perspectives of health professionals from several countries during a global pandemic. The study emphasizes the value of palliative care and identifies gaps in end-of-life care provision including resource allocation. This brings to light the urgent need to equip all health professionals with palliative care knowledge and skills, including discussing and planning care. Specialist palliative care teams are well placed to support health professionals to provide quality care.

## Introduction

The unparalleled impact of the COVID-19 pandemic has been experienced globally. Healthcare systems have been strained beyond their usual capacity as the widespread transmission of the illness has resulted in many individuals requiring significant medical treatment.^1^ The response to humanitarian crises, such as the COVID-19 pandemic involves saving lives and reducing suffering.^2^ The field of palliative care, which is well versed in relieving the suffering of individuals with life-limiting illnesses, has become part of a larger holistic response to such humanitarian crises.^2^ Palliative care is a human right with a focus on improving quality of life through the relief of physical, psychological, spiritual and emotional issues^3^ and the provision of support for complex decision-making to manage clinical uncertainty.^2^ Thus, palliative care is appropriately positioned to address the needs of individuals with COVID-19 who experience high symptom burden, distress and uncertainty during the end-of-life period.^1^

From the onset of the COVID-19 pandemic, health professionals around the world faced unprecedented changes in practice. Many were quick to share treatment ideas, experiences, and challenges in the form of online opinion articles and blogs, in which the Uniform Resource Locator (URL) to these were then posted on Twitter.^4^ Health professionals actively involved in providing palliative care used established online networks to share information, improve awareness of health issues, and discuss practice challenges and responses to the pandemic.^5,6^

Twitter, a communication platform, has been used previously in health science research as a source of data.^7^ However, to the best of our knowledge, no study to date has collected and collated online articles and blogs to explore health professionals’ perceptions of the role of palliative care during COVID-19. The aim of this study was to explore the role of palliative care as the COVID-19 pandemic unfolded, by analyzing the views and experiences of health professionals expressed in online opinion articles and blogs. The raw and personal views of frontline health professionals across numerous countries during a global pandemic are captured in this study through an approach of analyzing online articles and blogs which would not otherwise be captured through conventional qualitative data collection methods such as interviews and focus groups. Furthermore, Twitter was utilized to search for online opinion articles as the researchers observed a growing number of tweets containing URLs to online articles and blogs written by health professionals, as a means of sharing their experiences of care delivery during the COVID-19 pandemic.

## Methods

### Design

This qualitative exploratory study used naturalistic data^8^ in the form of online opinion articles and blogs. The opinion articles and blogs selected for this study were written by health professionals who documented their experiences and perspectives on the role and value of palliative care in the context of COVID-19 and subsequently shared the URL to these on Twitter. The consolidated criteria for reporting qualitative research (COREQ) guided the process of data collection and reporting.

### Data Collection

Data were collected over a 4-week period in July 2020. An online opinion article or blog was included in this study if: 1) the URL was posted on Twitter in the form of a tweet, 2) the online article was written between 1 February 2020 to 30 June 2020, to capture the timeframe when COVID-19 transmission was accelerating, 3) the online opinion article or blog was written by a health professional and 4) the article was related to palliative care in the context of COVID-19. Online opinion articles or blogs were identified using Twitter’s advanced search function to search for tweets containing a URL of relevant articles using 2 hashtags, #pallicovid and #COVID-19. These hashtags were identified via the free hashtag finder on Symplur™, a healthcare social media analytics tool, and were selected as they were the most frequently used hashtags related to the research topic. In Twitter’s advanced search function, additional key terms including “palliative care OR COVID-19 OR death OR dying” were utilized. The twitter posts were independently searched by 3 researchers (G.K.S, J.R., and J.F.) to locate relevant tweets containing the URL to online opinion articles and blogs, which were the source of data for this research. The opinion articles and blogs were between 448 and 2300 words, with a mean of 1037 words per article. The health professionals who authored the opinion articles were not known to the researchers. As such, the description given by the author of the opinion piece was used to determine that the author was a health professional.

### Data Analysis

Online articles were analyzed using content analysis for the purposes of gaining an in-depth understanding of the information being communicated.^9^ Excerpts from articles written by health professionals describing their views and experiences of palliative care in the COVID-19 environment were extracted and exported into a unified proforma Excel spreadsheet by each researcher. Details including date of publication, title, source, author/s, health discipline and expertise of the author (palliative or non-palliative) and country were extracted from the included online articles and blogs. Duplicates and articles that did not meet the inclusion criteria were checked and removed.

Data were analyzed through a constructivist lens to explore the experiences of health professionals in the context of COVID-19.^10^ The constructivist approach is consistent with the aim of understanding how health professionals interpret and are influenced by their social worlds, how they make sense of a situation from their experience and social interactions, and how this may impact the care they provide to patients during COVID-19.

Initial analysis involved familiarization with the data by reading the articles or blogs to achieve immersion and then re-reading to develop codes by highlighting words within the article or blog which illustrated key concepts of interest.^9^ Initial thoughts were noted, followed by development of the labels of the codes which were derived directly from the text.^9^ The codes were then arranged into categories according to their similarities and differences and the resulting categories were then grouped into analytical themes.^9^ The codes were created inductively and described the content of the data. All data were coded by a second researcher. Throughout the data analysis process, alternative interpretations, interrelations between codes and the development of themes were reviewed iteratively and discussed by the researchers to reach a consensus and ensure consistency regarding meaning and interpretation of the data. Both collection and analysis of the data occurred simultaneously to a point where sufficient, rich data were collected and no new concepts emerged.

### Ethics

Ethical approval for this study was obtained from Queensland University of Technology Human Research Ethics Committee (2000000447). The data were publicly available in the form of published online articles and blogs and no individuals were approached. As such, consent was waived. This study was conducted in accordance with principles embodied in the Declaration of Helsinki.^11^

## Results

### Demographic Profile of Health Profesionals

Thirty-three health professionals’ perspectives of the role of palliative care written in the form of articles or blogs, were included in the study. More than half of the health professionals were male (n = 19), most had a background in palliative care (n = 22) and 12 were specialist palliative care physicians. The health professionals were from Europe, North America, Oceania and Asia, with the largest group from the USA (n = 15). Demographics of health professionals are detailed in Table 1.

**Table 1.** Characteristics of Health Professionals.

	n	%
Sex		
Male	19	57
Female	14	42
Profession		
Palliative care background	22	
Chief medical officer	1	4.5
Family physician	1	4.5
Hospice nurse	1	4.5
Internal medicine specialist	1	4.5
Psychiatrist	1	4.5
Social worker	1	4.5
Chief of palliative medicine	2	9
Professor in palliative medicine	2	9
Specialist palliative care physician	12	55
Non palliative care background	11	
Acute care and trauma surgeon	1	7
Professor of medicine	1	7
Oncology nurse practitioner	1	7
Internal medicine physician	1	7
Intensive care physician	2	14
General practitioner	1	14
Emergency physician	1	14
Geriatrician	2	14
Family physician	1	14
Country of Author		
Australia	1	3
Italy	2	6
Germany	2	6
India	2	6
Scotland	3	9
Canada	4	12
England	4	12
USA	15	45

### Qualitative Findings

The key themes derived from content analysis centered on the perspective of palliative care during the pandemic. The themes were (a) the recognized value of palliative care during the pandemic (b) positioning palliative care skills as every health professional’s responsibility and (c) specialist palliative care supporting and guiding health professionals.

#### The recognized value of palliative care during the pandemic

The importance of improving the quality of life of all individuals with life-limiting illnesses, such as COVID-19, was emphasized during the pandemic. Palliative care services were instrumental in addressing the physical, psychological, and spiritual needs of patients and families.

“The importance of delivering effective palliative care as the COVID-19 epidemic unfolds is becoming more and more recognized.”^12^ (Non-palliative care background)

Palliative care teams have much to contribute during the pandemic with a focus on supporting complex decision making, managing clinical uncertainty and relieving suffering. Subsequently the need for palliative care services to respond and provide care is critical.

“There is no doubt about the value of palliative care as an approach in every clinical setting where corona patients are cared for and for specialized support where more complex multidimensional symptoms/problems occur.”^13^ (Palliative care background)

The ability of those with specialist palliative care skills to deliver care in accordance with patient preferences was deemed to be highly regarded by other health professionals.

“We find their recommendations helpful in offering adjunct or alternative care for the sickest of my patients, as their focus is often more relevant to what’s most important to the patient and to minimize their suffering.”^14^ (Non-palliative care background)

#### Positioning palliative care skills as every health professional’s responsibility

##### Increasing knowledge and skills of every health professional

As the pandemic continued, the provision of palliative care had become the responsibility of all health professionals.

“All doctors are palliative care doctors now.”^15^ (Non-palliative care background)

To avoid overloading specialist palliative care services, palliative care education and practice must be an essential component of generalist training. However, many medical trainees lack experience in palliative care. While palliative care has been highlighted as part of the response to COVID-19, many health professionals who provided care to patients during their end-of-life trajectory had not completed recognized competencies in palliative care.

“The majority of undergraduate and postgraduate medical trainees do not complete a palliative care clinical experience during their training, exposing an interesting dichotomy: although palliative care is an important part of the pandemic response, the majority of our physician workforce is not trained to provide it.”^16^ (Palliative care background)

The pandemic led to a call to expand the scope of practice of all medical, nursing and community care health professionals by including palliative care competencies during training to enable the delivery of basic palliative care. While the pandemic highlighted the need to increase palliative care training, if implemented, it is suggested that the benefits of palliative care training would continue in the post-COVID-19 healthcare environment.

“We hope that the tragedy of the Covid-19 pandemic will urgently promote training in palliative care and access to opioids for end of life care, bringing continuing benefit when the pandemic is over, but death continues.”^17^ (Palliative care background)

##### Involvement of all health professionals in complex decision making and goals of care discussions

In response to COVID-19, health professionals became increasingly involved in goals of care conversations and end-of-life discussions.

“This is a discussion you should have anyway, and so the coronavirus represents an opportunity to speak to your parents, grandparents, aunts, uncles and loved ones.”^18^ (Non-palliative care background)

During the COVID-19 pandemic, strategies to increase goals of care conversations included remote discussions and digital sharing of care plans between healthcare services. Telehealth appointments provided an innovative way to conduct goals of care conversations and patient-family interactions toward the end of life.

“Family doctors across the country have rapidly made telehealth appointments available, and advance care planning is a perfect use of this new resource.”^19^ (Non-palliative care background)

During the pandemic, health professionals became more proactive about goals of care conversations and end-of-life discussions with patients and families during the pandemic. The skills of specific groups of health professionals were also highlighted.

Surgeons must hone their skills in breaking bad news, disclosing prognosis (including prognostic uncertainty), and establishing goals of care.^20^ (Non-palliative care background)

#### Specialist palliative care supporting and guiding health professionals

Experiencing the extent of death and dying during the pandemic took an emotional toll on health professionals.

“I put my microphone on mute and cried…Often, I see fear in the eyes of those gasping for air and hear pain in the voices of adult children, asking me how long their mother or father has to live.”^21^ (Non-palliative care background)

Some doctors and nurses witnessed many deaths and others were forced to make decisions regarding the allocation of limited resources. This led many to question their ability to provide adequate care. Furthermore, they had little time to process their grief in the context of unfolding events.

“This means I may be faced with choices that, in my 20 years of clinical practice, I have never had to make…. These will be devastating decisions for all involved, including my colleagues.”^18^ (Non-palliative care background)

During the pandemic, specialist palliative care health professionals recognized the importance of supporting colleagues, both within and outside their specialist areas. In some situations, specialist palliative care clinicians were available via phone to coach primary care teams. Supporting and empowering colleagues who had encountered rapidly changing clinical roles, and increased risks to their own health, was vital during the COVID-19 pandemic.

“This pandemic means being ready to support hard-working colleagues as they face risks to their own health on top of the emotional toll that overstrained resources, death and tough conversations can bring to us all.”^22^ (Palliative care background)

Some specialist palliative care health professionals recognized their ability to assist and support other health professionals beyond their regular interdisciplinary team.

“We can help our health system understand how to collaborate with the wisdom embedded in our communities—not only the doctors, nurses, social workers, and chaplains we often work but even beyond. It is time to expand our interdisciplinary team.”^23^ (Palliative care background)

Additional quotations are provided in supplemental material.

## Discussion

The COVID-19 pandemic has underlined the value of palliative care and the need for it to be the responsibility of all health professionals. Furthermore, the pandemic has highlighted that health professionals require education in palliative care competencies. Specialist palliative care professionals are also well placed to support non-palliative care trained health professionals.

This study highlights that some health professionals felt that they do not have the skills required to provide quality end-of-life care and require more education and training in order to do so. This gap in practice can be addressed by equipping health professionals with skills to conduct goals of care conversations and improving their knowledge of palliative care. This may help to alleviate the existing demand for palliative care due to a shortage of a specialist palliative care physicians.^24^ Current evidence suggests that the healthcare workforce does not receive adequate palliative care education during medical training.^25,26^ This viewpoint is shared by students who suggest that medical schools are not adequately preparing future health professionals to conduct end-of-life care conversations.^27^ The inherent emotionality, stigma and taboo surrounding death and dying is a challenge.^27^ As a priority, undergraduate health professional courses should prepare graduates to effectively communicate topics surrounding grief and loss, existential issues and changing goals of care.^28^ In addition, health professionals need to appreciate the diverse clinical responses that occur throughout illness trajectories, to understand the palliative principles for assessing and managing care needs and the ability to self-reflect on one’s own professional and personal experiences.^28^ The experiences of health professionals, as reflected in this study, highlight the need for increased palliative care education for the emerging and current healthcare workforce.

Health professionals in this study value the importance of discussing and planning care as a means of reducing patient and family distress during COVID-19 and in facilitating patient-centered care. COVID-19 has highlighted that the benefit of goals of care conversations is derived from the conversations that make up the planning process, rather than a finalized document.^29^ Goals of care conversations is an iterative process and should aim to identify a patient’s understanding of their medical issues, assess their desire to receive information, consider their goals and values and provide time for reflecting with family.^30^ While goals of care conversations were viewed as important to improve patient care, findings from this study suggest health professionals have a gap in knowledge and skills relating to leading goals of care conversations and the provision of palliative care; an area in which the specialist palliative care workforce is well trained. Specialist palliative care clinicians can foster communications, training and support to non-specialist palliative care health professionals during the COVID-19 pandemic. Palliative care educational endeavors should prioritize the development and dissemination of resources for non-specialist palliative care teams around communication skills.^31^

The COVID-19 pandemic has impacted the health and wellbeing of health professionals. This is in accordance with recent studies demonstrating that health professionals providing care to patients with COVID-19 experienced high levels of emotional exhaustion and depersonalization,^32^ a consequence of an overwhelming workload and inadequate support.^33^ Emotional distress experienced by health professionals requires urgent attention as major unaddressed distress can result in patients receiving suboptimal care, in addition to long-term negative effects on health professionals’ well-being.^34^ These stressors may be alleviated by providing additional guidance, resources, and training.^35^ Specialist palliative care health professionals have acknowledged their role in providing support and reassurance to colleagues, both within and outside of their specialist area, who may experience feelings of overt responsibility for the deaths of patients. This has been achieved by specialist palliative care services developing support packages that provide information and guidance.^36^ Furthermore, tools and telephone support,^37^ to enable non-specialist palliative care health professionals to deliver holistic palliative care have been provided. These resources aim to empower and support all health professionals to deliver high-quality non-specialist palliative care during the COVID-19 pandemic and highlights the importance of specialist palliative care supporting non-specialist palliative care health professionals beyond the pandemic.

### Strengths and Limitations

A strength of this study was using Twitter’s advanced search function and including keywords and hashtags to search for relevant online articles and blogs posted on Twitter. Locating online opinion articles and blogs on Twitter is a useful mechanism for locating temporally relevant information related to individuals and events. This study utilized naturalistic data which contain raw and personal experiences of health professionals and reduces the effect of biases or actions of the researchers. There was over-representation of health professionals with a palliative care background. This may be the result of these individuals being more likely to share their perspectives of palliative care during the COVID-19 pandemic. The study included the perspectives of health professionals from a range of anglophone countries. This may not capture global nuances of health professionals’ perspectives toward palliative care during COVID-19. It does, however, provide a rich record of individuals viewpoints that provide insight into the role of palliative care during the pandemic.

## Conclusion

Online opinion pieces and blogs shared on social media have been a means to bring clinicians together to learn from and support each other during the COVID-19 pandemic. The pandemic has provided a lens that highlights a need to rapidly expand the capacity of all health professionals to deliver non-specialist palliative care. The COVID-19 pandemic has also afforded an opportunity for health professionals to be proactive in goals of care conversations. Specialist palliative care teams are well placed to support and guide other health professionals, as the pandemic has highlighted the need to ensure that all patient care is compassionate and goal concordant. It is essential that all health professionals are supported and empowered as they continue to make sense of the current pandemic and prepare for what might lie ahead.

## Supplemental Material

Supplemental Material, sj-pdf-1-ajh-10.1177_10499091211024202 - Health Professionals’ Perspectives of the Role of Palliative Care During COVID-19: Content Analysis of Articles and Blogs Posted on Twitter Click here for additional data file. Supplemental Material, sj-pdf-1-ajh-10.1177_10499091211024202 for Health Professionals’ Perspectives of the Role of Palliative Care During COVID-19: Content Analysis of Articles and Blogs Posted on Twitter by Gursharan K. Singh, Joanna Rego, Shirley Chambers and Jennifer Fox in American Journal of Hospice and Palliative Medicine®
